# Optimized minimal genome-wide human sgRNA library

**DOI:** 10.1038/s41598-023-38810-6

**Published:** 2023-07-18

**Authors:** Yangfan Zhou, Lixia Wang, Zhike Lu, Zhenxing Yu, Lijia Ma

**Affiliations:** 1 College of Life Sciences, Zhejiang University, Hangzhou, 310058 China; 2 School of Life Sciences, Westlake University, 600 Dunyu Road, Hangzhou, 310030 Zhejiang China; 3 Institute of Biology, Westlake Institute for Advanced Study, Hangzhou, 310024 Zhejiang China; 4 Westlake Laboratory of Life Sciences and Biomedicine, Hangzhou, 310024 Zhejiang China; 5 School of Life Sciences, Fudan University, Shanghai, 200433 China

**Keywords:** Biotechnology, Genetics, Molecular biology

## Abstract

Genome-wide clustered regularly interspaced short palindromic repeats (CRISPR)-based knockout screening is revolting the genetic analysis of a cellular or molecular phenotype in question but is challenged by the large size of single-guide RNA (sgRNA) library. Here we designed a minimal genome-wide human sgRNA library, H-mLib, which is composed of 21,159 sgRNA pairs assembled based on a dedicated selection strategy from all potential SpCas9/sgRNAs in the human genome. These sgRNA pairs were cloned into a dual-gRNA vector each targeting one gene, resulting in a compact library size nearly identical to the number of human protein-coding genes. The performance of the H-mLib was benchmarked to other CRISPR libraries in a proliferation screening conducted in K562 cells. We also identified groups of core essential genes and cell-type specific essential genes by comparing the screening results from the K562 and Jurkat cells. Together, the H-mLib exemplified high specificity and sensitivity in identifying essential genes while containing minimal library complexity, emphasizing its advantages and applications in CRISPR screening with limited cell numbers.

## Introduction

CRISPR/Cas technology offers a versatile toolbox for genome editing. Facilitated by the efficiency of Cas9 endonuclease, specific genes can be targeted and disrupted simply by changing the sequence of the single guide RNA (sgRNA), which leads to the generation of genome-wide CRISPR-based knockout screening^1,2^. These CRISPR-based knockout screening systems have been applied to hundreds of cell types in different organisms^3,4^ and made significant advancements in unraveling biological processes in many aspects, for example, functional genomics^5^, cancer, and immunotherapy^6–9^. Accordingly, many studies have been performed to improve the performance of CRISPR-based knockout screening systems, including CRISPR/Cas enzyme optimization^10^ sgRNA library design^11–13^, and sgRNA delivery^14^. However, the sgRNA library size remains a stringent barrier to the application of CRISPR-based knockout screening systems.

The sgRNA library size limits the development of CRISPR-based knockout screening technology from several aspects: (1) Full library representation needs typically around 50–100 × the original size of the library^15^. In the typical workflow of CRISPR-based knockout screening, most cells possess mutations in only one gene, meaning one sgRNA for one cell. However, from the moment the library is introduced into the cell, the sgRNA distribution will immediately be affected by the culturing process and downstream processing which may lead to a loss of yield and potential representation. Therefore, each sgRNA must be represented by 50–100 cells to mitigate this. Finally, the screening cell population will be 50–100 × larger than the sgRNA library. (2) Unfortunately, some cell populations can’t reach millions of cell numbers easily for whole genome screening, as primary cells and non-immortalized lines have a limited capacity for expansion. (3) Additionally, a larger library size means more cost. The manipulation size for experiments of sgRNA pool construction, the delivery system establishment, and cell treatment will increase exponentially on the original sgRNA library number, leading to exponentially increased consumption. (4) Moreover, the sgRNA number is typically 4–10 × larger than the number of target genes^11–13,15,16^. sgRNA library is designed with inherent redundancies to reduce sgRNA off-target effects and achieve equal representation and performance across all target genes. Therefore, the redundant sgRNAs library is widely used and challenges the feasibility of a CRISPR-based knockout screening system.

Recently, some genome-wide human sgRNA libraries have been assembled. Especially for mouse and human cells, several libraries are now widely available in public plasmid repositories^3,4,17,18^. For example, Brunello^19^, Gattinara^20^, GeCKOv2^21^, and TKOv3^22^. Those libraries are designed with different rules, contain varying numbers of sgRNAs and target genes, and are for distinct applications. In this research, we designed the minimal human genome-wide sgRNA library (H-mLib), by utilizing a dual sgRNA CRISPR/Cas system with novel selection strategies. The performance of H-mLib was validated by K562 cell fitness screening. Screening results demonstrated the outperforming specificity and efficiency of the H-mLib library, guaranteeing the reliability and feasibility of H-mLib for further application. Combined with Jurkat cell fitness screening, we expanded the human core-essential gene list and defined cell-type specific essential genes.

## Results

**Figure 1:**
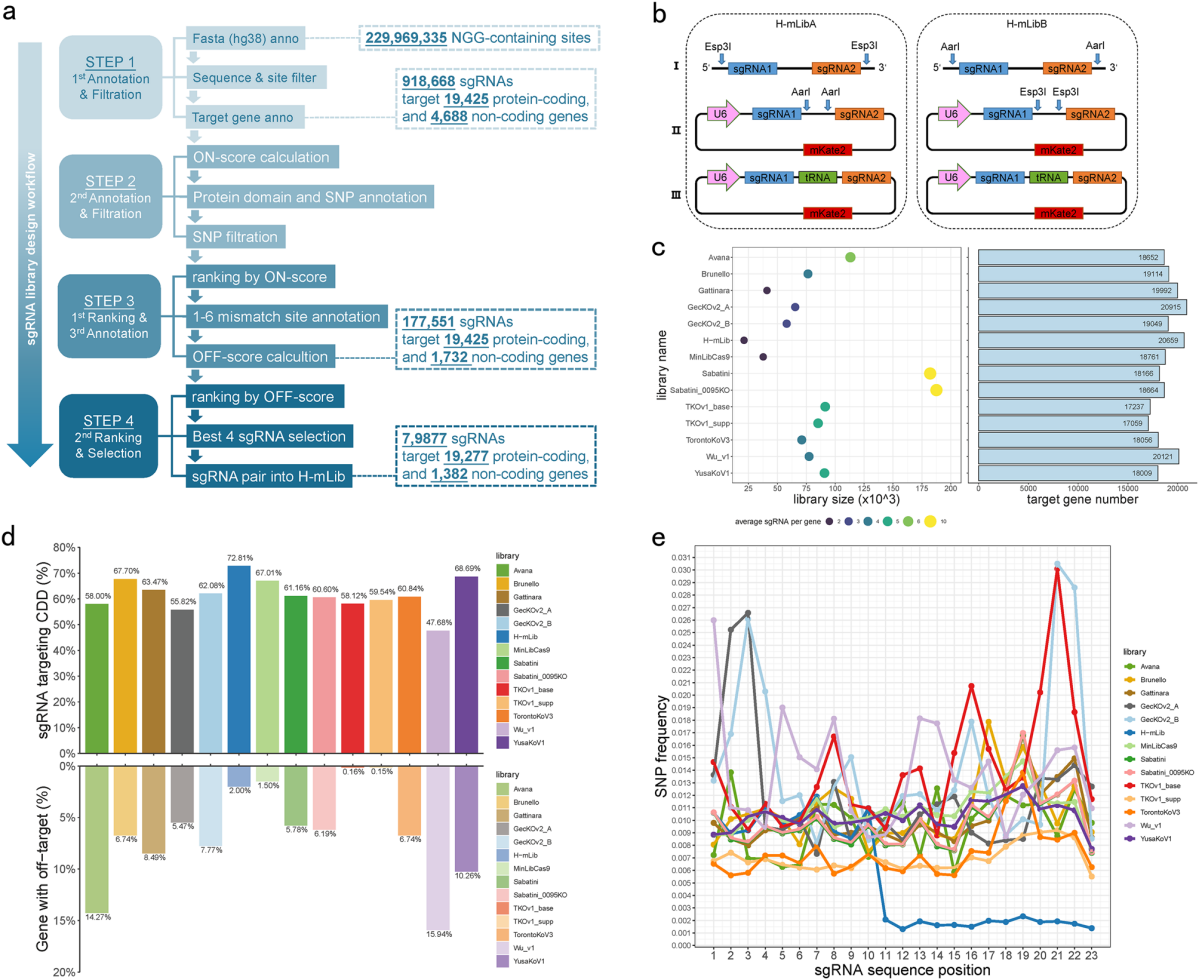
Design and property of H-mLib sgRNA library. (**a**) sgRNA design workflow of H-mLib sgRNA library. The process involves iterative annotation and filtration of candidate sgRNAs to identify the most effective sgRNAs. The final selected sgRNAs and their target genes after the iterative process were shown in the dotted box. (**b**) Constructs and schematic illustration of the dual-sgRNA system used by H-mLibA (left) and H-mLibB(right). **I** Synthesized oligonucleotide of H-mLibA and H-mLibB, each oligonucleotide contains two sgRNAs. **II** The construct of sgRNA oligo and the backbone plasmid which contains U6 promoter and expresses mKate2. According to the utilization of opposite restriction endonuclease, sgRNAs could clone into a specified plasmid. **III** Human transfer RNA Gln (tRNA-Gln) was constructed into the plasmid too, the tRNA processing system allows pairwise sgRNA expression in a single cell. (**c**) Library size and target gene number of H-mLib and other reported CRISPR/Cas9 libraries. The name of each sgRNA library is shown vertically on the left and the corresponding number of target genes is displayed in the histogram on the right horizontally. The library size and average sgRNA number per gene were shown in dot plot on the left, corresponding to library names horizontally. (**d**) CDD target rate and gene off-target rate of H-mLib and other reported CRISPR/Cas9 libraries. The bar plot on the top shows the percent of sgRNAs target CDD region in each sgRNA library. The bar plot on the bottom shows the percent of genes containing sgRNAs that may have off-target sites on the genome. (**e**) SNP frequency at each site of sgRNA (1–20) and PAM (21–23) sequence in H-mLib and other reported sgRNA libraries. The lower SNP frequency at position 11–23 of H-mLib contributes to the lower off-target possibility.

To minimize the number of required sgRNAs while maximizing the number of targeted genes, we evaluated and selected sgRNAs from all potential sgRNAs with NGG PAMs in the human genome, which yielded 229,969,335 sgRNA sequences. To increase the targeting specificity, we removed sgRNAs containing repetitive polynucleotide stretches or targeting more than six (≥ 6) genomic sites (Fig. 1a; “Methods” section). We obtained a Primary Pool of 918,668 sgRNAs covering 19,425 protein-coding genes and 4688 non-coding genes. To evaluate the on-target efficiencies of each sgRNA to further filter sgRNAs, we conducted the on-target efficiency prediction using algorithms from multiple kinds of literature, including Project score^22^, Rule2 score^19^, DeepCas9 score^23^, and AIdit_ONs score^24^. We found that the correlations of these scores varied across different prediction algorithms (Supplementary Fig. 1), which might result from different calculation procedures and emphasis on sgRNA design principles. We then used a weighted sum of the above four scores to eliminate the bias from the individual algorithm to integrate these scores into our sgRNA selection pipeline, and the resulting sum was named ON-score (Fig. 1a; “Methods” section). To evaluate the ON-score performance in predicting the on-target efficiency, we used thirty-two public datasets^25^ by comparing the experimentally measured cleavage efficiency of each sgRNA to multiple prediction scores (Supplementary Fig. 2). Across the datasets, the ON-score showed higher correlations in two-third of cases compared to the other four scores (Supplementary Fig. 3) and was employed to evaluate gRNAs in the Primary Pool for further selection.

Besides the ranking based on the ON-score, we also considered the location of sgRNAs within the targeting gene under the context of biological indication and sequence polymorphism. It has been recognized that conserved domains largely contributed to a protein’s cellular and molecular function^26^. Thus, we reasoned that sgRNA targeting the genomic sequence of the conserved domain may perform better than targeting other regions of a gene in a dropout screening by interrupting the essential function of that protein. Moreover, we also considered the influences from the reported single-nucleotide polymorphism (SNP), which may eliminate the efficiency of hybridization between the designed sgRNA sequence and the targeting genomic region, especially when SNPs occurred near the PAM region^27,28^. Together, the following two criteria were applied to further select sgRNAs for each gene from the Primary Pool: (1) the cutting site of each sgRNA located in the conserved domain annotated by the conserved domain database (CDD)^26^; and (2) the sgRNA sequence only contains SNP at distal-to-PAM region (10 nucleotides at the most 5’ of sgRNA). Finally, the top 10 sgRNAs after ON-score ranking and filtering were retained for each gene. As a result, we got 177,551 sgRNAs covering 21,157 genes (Fig. 1a; “Methods” section).

Besides the on-target editing efficiency, off-target editing specificity is also essential to the performance of sgRNA. To further evaluate the editing specificity of sgRNAs, we employed the cutting frequency determination (CFD) score^19^ to calculate the number of potential off-targets in the human genome, which were used as the OFF-score in our sgRNA selection pipeline (Fig. 1a; “Methods” section). Up to six mismatches were allowed in CFD score calculation for the interests of computing resources. By incorporating the OFF-scores, we further selected the best four sgRNAs of each targeting gene and split them into two parallel minimal whole-genome libraries, with the top two sgRNAs composed of the H-mLibA and the other two sgRNAs composed of the H-mLibB. In some cases, when less than four sgRNAs are available for one target gene, sgRNAs were shared between the H-mLibA and H-mLibB (Supplementary Fig. 4a, b; “Methods” section). Each of these two parallel libraries composed a minimal set of the best-performed sgRNAs targeting the full set of human genes, which could also be used as independent replicates in the whole-genome CRISPR screening.

To further minimize the complexity of the minimal library and increase the screening performance, we designed a dual-sgRNA vector to accommodate the two sgRNAs of each targeting gene (Fig. 1b). We also designed two compatible cloning strategies, in which the sgRNA libraries of the H-mLibA and H-mLibB could be synthesized as one oligo pool while the plasmids libraries could be cloned separately. Each plasmid library sized at 21,159 complexities contained 20,659 sgRNA pairs and 500 negative controls (Supplementary Table 1). Compared to the published libraries (Avana^19^, Brunello, Gattinara, GecKOv2_A, GecKOv2_B, MinLibCas9^29^, Sabatini^30^, Sabatini_0095KO, TKOv1_base^3^, TKOv1_sup^3^, TKOv3, Wu_V1^31^ and YusaKoV1^32^), the H-mLib advantaged with the minimal library size and the second-highest number of targeted genes (Fig. 1c). Benefited from the sgRNA selection strategies with the considerations on the genomic location, the H-mLib also showed the highest targeting rate (72.81%) to the conserved domain^26^, while the low percentage of genes contain sgRNAs that may have off-targets in the genome (Fig. 1d). Additionally, compared to the other libraries, the sgRNA of the H-mLib showed significant lower SNP frequency from the positions of 11–20 and 21–23 (PAM sequence) (Fig. 1e).

Meanwhile, we examined the consistency of selected sgRNAs between H-mLib and published libraries. We incorporated sgRNAs from the GenomeCRIPSR database^33^, as it collected approximately 700,000 sgRNAs used in ~ 500 different experiments. Most sgRNAs of the H-mLib have been included, but there were 805 sgRNAs unique to the H-mLibA and 672 sgRNAs unique to the H-mLibB (named “sup”) (Supplementary Fig. 5). We further investigated the property of these “sup” sgRNAs and compared them with those targeting the same gene in other libraries. The median ON-score calculated for these sgRNAs in H-mLib was higher than the most of other libraries (n = 11), while only Gattinara and TorontoKoV3 show comparable distributions (Supplementary Fig. 6). Meanwhile, the percent of sgRNA targeting CDD got similar results, only Brunello, Gattinara, and YusaKoV1 were a little bit higher than H-mLib (Supplementary Fig. 7). Moreover, the SNP frequency at each position of “sup” sgRNAs in H-mLib is much lower than in any other libraries, and in some positions, there is no SNP that could guarantee the on-target efficiency (Supplementary Fig. 8). These results suggest that the existing genome-wide CRISPR/Cas9 libraries still have room for optimization and may miss some sgRNA candidates.

**Figure 2:**
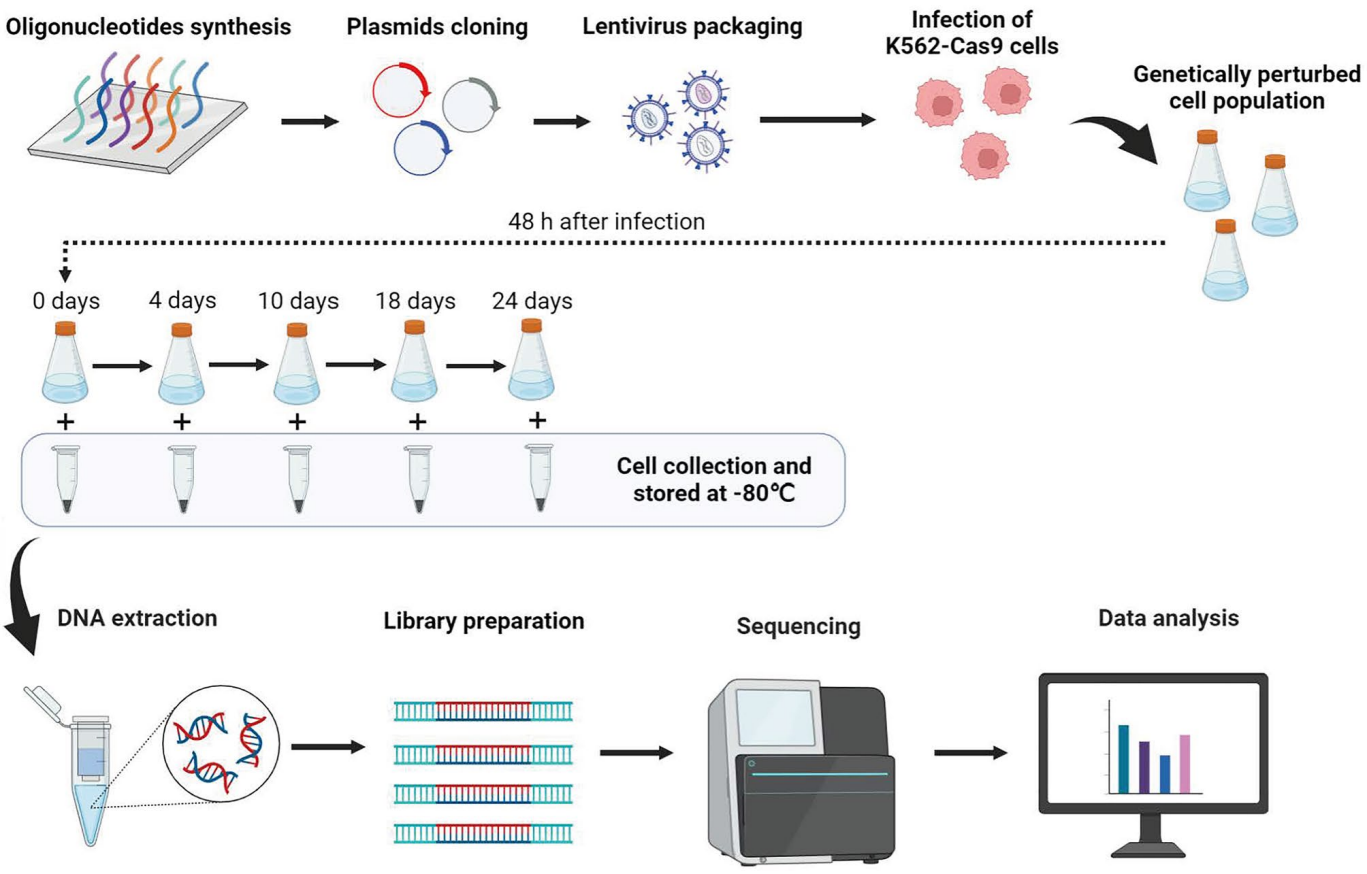
Schematic of K562 CRISPR/Cas9 knockout screening using H-mLibA, H-mlibB, and Brunello libraries. The oligonucleotides of H-mLib and Brunello containing a different number of sgRNA were cloned into plasmids and transduced into K562 cells through lentivirus. Cells were collected at five different time points. Benefiting from the small library size and dual-sgRNA system, the number of plasmids and viruses required for H-mLibAand H-mLibB were four times less than Brunello’s.

**Figure 3:**
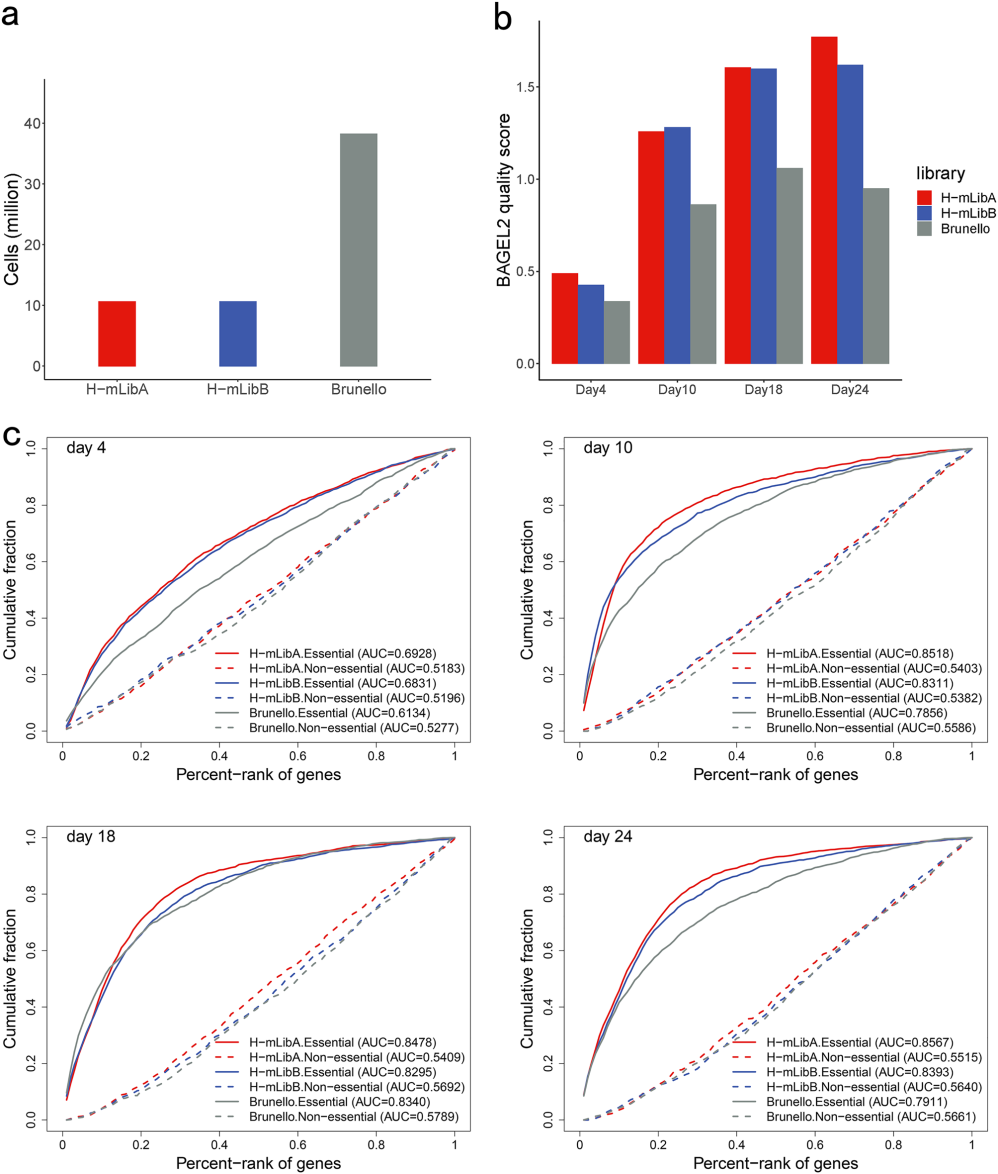
Genome-wide CRISPR/Cas9 knockout screens in the K562 cell line. (**a**) Required cell number for H-mLibA, H-mLibB, and Brunello libraries screening. To reach a 500 × coverage screening experiment, H-mLibA and H-mLibB require approximately 11 million cells while Brunello requires approximately 38 million. (**b**) Mean quality score of H-mLibA, H-mLibB, and Brunello libraries on different time points. The quality scores of replicates were directly related to the overall reliability of an experiment. H-mLibA and H-mLibB have the same quality scores across all time points and are much higher than Brunello after Day 10. (**c**) ROC-AUC analysis of individual sgRNAs targeting essential (solid line) and non-essential (dashed line) gene sets in the H-mLibA, H-mLibB, and Brunello library screened in K562 cells at time point day 4, day 10, day 18 and day 24. The results were calculated by MAGeCK.

Next, we conducted a fitness CRISPR screening in K562 cells to validate the performance of the H-mLib sgRNA library (Fig. 2; Supplementary Table 2). We chose the Brunello library as a benchmark as it has been widely used in many CRISPR-based knockout screening experiments^11,27,34–38^ since it was designed. And when comparing the prediction scores of on-target efficiencies, the Rule2 score of the Brunello library showed the second-best performance besides H-mLib (Supplementary Fig. 2; Supplementary Fig. 3). In brief, the sgRNAs of the H-mLibA and the H-mLibB libraries were synthesized and cloned into a dual-gRNA vector respectively, while the Brunello library was cloned into a single-gRNA vector according to its original design. The three plasmid libraries were then transduced into K562 cells, which were cultured for 24 days with the presence of puromycin (“Methods” section). To achieve the 500 × cell-to-sgRNA coverage, each of the H-mLib libraries requires about 11 million cells and the Brunello library requires 38 million cells (Fig. 3a). The quality control analysis of the plasmid library before lentivirus vector packaging and the “0 day” reference library (48 h after transduction) revealed consistent and uniform characteristics. The correlation between the plasmid library and the day 0 library was found to be greater than 0.88, indicating that the sgRNA abundances in the virus library align with those in the plasmid library. Additionally, the quality control assessment demonstrated high coverage rates of over 99% and 95% for the plasmid library and day 0 library, respectively. The coverage was also observed to be uniform, with a 90/10 ratio of less than 2 and 2.5 for the plasmid library and day 0 library, respectively (Supplementary Fig. 9 and 10). K562 cells bearing the CRISPR screening libraries were collected on day 4, day 10, day 18, and day 24, and NGS libraries were prepared to quantify the sgRNAs abundances in these cells.

To determine the overall performance of our screening experiments, we employed a comprehensive essential gene list which was summarized by six gene lists (“Methods” section) and a gold-standard nonessential gene list^39^ as reference gene sets. We first use the replicate quality scores calculated by BAGEL2^40^ to evaluate the reliability of screens. Along with the screening, the quality scores increased accordingly across all three libraries, and after day 4 both the H-mLibA and H-mLibB showed reliable quality replicate (quality score > 1). Besides, H-mLibA and H-mLibB got higher score results than Brunello at any time point (Fig. 3b).

At the gene level, we performed Receiver Operating Characteristics (ROC)—Area Under The Curve (AUC) analysis on individual sgRNAs, which is a measurement of the classification performance on essential and non-essential genes and the AUC values represent the degree or measure of separability^41^. According to the MAGeCK^42^ results, we plotted the ROC curve to estimate the performance of each library (Fig. 3c; “Methods” section). After day 4, all libraries showed high performance in the detection of essential genes while both H-mLibA and H-mLibB performed better than Brunello. For non-essential genes, all libraries showed a random distribution (AUC ~ 0.5) which indicated these genes were not preferentially depleted. The results showed the AUC value of all libraries increased remarkably from day 4 to day 10 and remained at a similar level till day 24. Notably, the H-mLibA and H-mLibB reached a high AUC value (0.8518 and 0.8311) on day 10, which is earlier than day 18 of the Brunello. Moreover, the H-mLibA showed higher AUC values than Brunello across all time points for essential genes. Furthermore, we employed two additional algorithms, ScreenBEAM^43^ and PBNPA^44^ to do the same analysis (Supplementary Fig. 11; “Methods” section). Although different algorithms got variable results, in all the circumstances, H-mLibA and H-mLibB performed remarkably well than Brunello.

Genome-wide pooled libraries display possibilities to identify and classify essential genes in different species, tissues, or cell types. These essential genes are likely related to different biological functions and processes and can be classified into core-essential and tissue/cell-specific essential genes. By identifying the gene essentiality, we could gain new insights into key cellular processes and find important targets for disease therapies^3,4,17,45^. To identify the essential genes, the H-mLib and Brunello libraries were also used to screen Jurkat cells, and cells were collected at the effective time points (day 12, day 18, and day 24) refer to K562 (Supplementary Table 3). The screen results of Jurkat cells showed high screening performance as good as K562 (Supplementary Fig. 9, 11, 12, and 13). Then we applied the Bayesian analysis of gene essentiality approach to calculate a log Bayes factor (BF) for each gene, and genes with BFs above a certain threshold were considered as essential genes (“Methods” section). In the end, the essential genes overlapping by K562 and Jurkat were identified as core-essential, and other genes unique from any given essential gene lists were identified as cell-specific essential. As a result, we identified 211 human core-essential genes, which contain 132 genes overlapping the previously defined set^45^ and 79 additional genes (Supplementary Fig. 14). Furthermore, we identified 16 K562 and 12 Jurkat cell-type specific essential genes which were uniquely classified (Supplementary Table 4; “Methods” section). For K562 cell-type specific essential genes, we observed feline leukemia virus subgroup C cellular receptor 1 (FLVCR1), which has been suggested to relate to erythropoiesis^46^, and GATA binding protein 1 (GATA1), which functions in erythroid development^47^ and biased expression in bone marrow^48^. For Jurkat cell-type specific essential genes, we observed menin 1 (MEN1), which was related to a tumor suppressor^49^. These results would be valuable for identifying specific therapeutic targets. Together, the H-mLab had outperformed efficiency on not only gene knock speed but also total gene deletion rare. Moreover, the minimal library size offers the greatest cost savings and best expandability in the future.

## Discussion

CRISPR-based knockout screening is an emerging technology that enables systematic genetic analysis of a cellular or molecular phenotype in question. Here, we design an optimized minimal genome-wide human sgRNA library, termed H-mLib. Comparative data indicate that H-mLib has the smallest library size, yet second-largest targeting gene numbers (Fig. 1c). The library size has been minimized through an improved sgRNA design strategy and utilizing a dual-gRNA delivery system, in which two sgRNAs are employed to target a single gene, significantly contributing to reducing the library size.

To design the dual-sgRNA, we gave priority to ensuring that sgRNA1 is the one with the highest efficiency, unless the prioritized sgRNA positions are conflict with restriction sites of the cloning steps. Previous studies have demonstrated that in the tRNA-sgRNA system, the position of sgRNA has no significant impact on the editing efficiency, suggesting the dual-sgRNA design would not lead bias to the final screening outcomes. It has also been reported that the distances between the sgRNA targets may induce different efficiency of deletion^50,51^. However, we did not prioritize the distances between the sgRNA1 and sgRNAs as this will significantly decreased the number of available sgRNAs of each gene (Supplementary Fig. 15).

In the fitness screening performed in K562 and Jurkat cells, both H-mLibA and H-mLibB demonstrated better library quality and screening efficiency compared to the Brunello library (Fig. 3; Supplementary Fig. 11, 12, and 13), and they also exhibit superior performance in gene knock speed and total gene deletion rate (Supplementary Fig. 16). The paralleled screenings allowed us to analyze core essential genes and cell-type specific essential genes in K562 and Jurkat cells. Among the 211 essential genes we identified from the K562 and Jurkat screening, 132 genes were overlapped with CEGv2^45^ and 79 were new from this study (Supplementary Fig. 14). Meanwhile, we set up a series of cutoffs and identified 16 and 12 cell-type specific essential genes in the K562 and Jurkat cell lines, which may provide valuable insights for targeted therapeutic interventions.

The size of the H-mLib allows screening when the number of cells are limited, and the smaller screening scale also saves cost. According to the K562 and Jurkat fitness screening, the H-mLib also demonstrated good screening efficiency, reflected by dropping out of genes in earlier time points than other libraries.

In conclusion, the minimal-sized and efficient sgRNA library, H-mLib, added a valuable module to the CRISPR screening toolbox and provided more opportunities to identify critical genes in biomedical researches.

## Methods

### sgRNA collection and enzyme cut site annotation

All sgRNAs corresponding to potential NGG-containing target sites on the human (GRCh38/hg38) genome were calculated by a customized Perl script. The enzyme cut site was set at the 17 position from 5’ to 3’ of each sgRNA sequence. These sgRNAs have more than six (≥ 6) target sites or contain more than four (≥ 4) consecutive bases that were filtered out first. Bedtools^52^ 2.30.0 was used to annotate the enzyme cut site of each sgRNA according to the overlap between cut coordinates with human genome annotation files downloaded from the National Center for Biotechnology Information (NCBI) database^53^. For genes without any sgRNA annotation, we search sgRNAs with no more than ten (≤ 10) target sites and retain those that could be annotated to a single gene. In the end, a primary pool of 918,668 sgRNAs was generated.

### ON-score calculation

DeepCas9 score program takes a 30 bp sequence (4 bp upstream + 20 bp target + 3 bp PAM + 3 bp downstream) as an input file. The DeepCas9 package is carried out with default parameters^23^. AIdit_ONs score algorithm takes a 63 bp sequence (20 bp upstream + 20 bp target + 3 bp PAM + 20 bp downstream) as an input file to predict the on-target activities^24^. The selected cell type and genome editing enzyme were K562 and SpCas9 respectively. The Project score only requires a 20 bp sgRNA sequence and simply adds the nucleotide scores at each position according to the score table^45^. Rule2 score program takes a 30 bp sequence same with DeepCas9 as input and only the “–seq” parameter is set^11^. After Z-score normalization, Pearson’s correlation was calculated and the highest correlation coefficients were set as score weights for each score. The final ON-score was the sum of the weighted score of AIdit_ONs score, DeepCas9 score, Project score, and Rule2 score.

### Ranking of sgRNA and OFF-score calculation

All sgRNAs in the primary pool are assigned to each gene according to their target sites. Besides, SNPs and conserved domains are also annotated by the same method of enzyme cut site annotation. The human SNP annotation file was downloaded from the NCBI database. Conserved domain annotation information was downloaded from the Prot2HG database^54^. sgRNAs are removed if any SNP sites were deposited in positions 11–20 (5′–3′) or in the PAM sequence. Then the remaining sgRNAs of each gene are sorted in descending order according to the ON-score, and the conserved domain target sgRNAs will be carried out first. Finally, the top 10 sgRNAs were selected, and all sgRNAs were selected if the total number of sgRNA is less than 10.

Next, the potential off-target sequences of these sgRNAs with 1 to 6 mismatches are collected by searching all NGG-containing sequences on the human genome. These sequences were used to calculate CFD scores by the CRISPOR^55^ tools with default parameters and the OFF-score is the sum of all CFD scores for each sgRNA.

### Minimal sgRNA library generation

The top 10 sgRNAs were divided into two sgRNA groups according to the enzyme cut site number: unique target and multiple target. In the next sgRNA selection step, the unique target group is prioritized. Within each subgroup, sgRNAs are sorted by OFF-score in ascending order. After filtering out the recognition sites of BsmBI and AarI, the best four ranked sgRNAs were selected and constructed into two separate dual knock-out libraries, namely H-mLibA which contains the top two sgRNAs, and H-mLibB which contains the remaining two. If there are not enough sgRNAs, the best-ranked one will be reused. In the two libraries, we added the same 500 non-target 20-nt sequences on the human genome as the negative control.

### Lentiviral vectors construction

Oligonucleotides of the H-mLib library were synthesized by GenScript (CustomArray) and amplified in six 50 ul PCR reactions as follows: 25 ul NEBNext Ultra II Q5 Master Mix (NEB, M0544S), 2.5 ul forward primer (10 uM), 2.5 ul reverse primer (10 uM), 1 ul template (5.33 ng/ul oligo pool), and nuclease-free water up to 50 ul. The PCR program of H-mLibA was set as the following condition: (1) 98 °C 30 s; (2) 12 cycles of 98 °C 10 s, 64 °C 30 s, 72 °C 30 s; (3) 72 °C 2 min. The PCR program of H-mLibB was set as the following condition: (1) 98 °C 30 s; (2) 12 cycles of 98 °C 10 s, 70 °C 30 s, 72 °C 30 s; (3) 72 °C 2 min. The PCR products were size separated on 2.5% agarose gel. The gel slice with the targeted size (115-nt) was extracted using the QIAGEN Gel Extraction kit (QIAGEN, 28706) and further purified with 1.8 × AMPure XP beads (Beckman, A63882). The purified products were cloned into a modified lentiGuide Puro backbone (addgene, 52963) with mKate2. All primers used here can be found in Supplementary Table 5 (Supplementary Table 5).

The 50 uL Golden Gate Assembly (GGA) reaction of H-mLibA was set as follows: 50 fmol of backbone, 150 fmol of inserts, 0.5 ul of T4 DNA ligase (Thermo, EL0014), 5 ul 10 × T4 DNA ligase buffer, 1 ul Esp3I (Thermo, ER0451), and nuclease-free water up to 50 ul. The 50 uL GGA reaction of H-mLibB was set as follows: 50 fmol of backbone, 150 fmol of inserts, 0.5 ul of T4 DNA ligase (Thermo, EL0014), 5 ul 10 × T4 DNA ligase buffer, 1 ul Esp3I (Thermo, ER0451), and nuclease-free water up to 50 ul. The GGA condition of each library was set as (1) 90 cycles of 37 °C 5 min and 22 °C 5 min; (2) 65 °C 30 min; (3) 37 °C 3 h. For H-mLibA and B, an additional 1 ul of Esp3I and AarI was added to the reaction right before the 3 h 37 °C incubation, respectively. For each library, three 50 ul reactions and one negative control reaction were performed following the same condition except without adding the inserts. The GGA reaction products were purified with 0.8 × AMPure XP beads (Beckman, A63882) and then dialysis on the MFMillipore™ Membrane Filter (Sigma, VSWP02500) for 2 h. For each transformation reaction, 2 ul GGA products were electroporated (Eppendorf 2510, 1700 V) with 25 ul electrocompetent cells (Lucigen, 60242-2). One reaction was performed for the sample and one reaction was performed for the negative control. The tube with the transformation mixture was recovered for 1 h at 37 °C, then spread on two 25 cm × 25 cm LB-ampicillin plates and incubated for 20 h at 30 °C. After propagation, colonies were scraped from the plates. Plasmids were extracted using QIAGEN Plasmid Plus Midi Kit (QIAGEN, 12945) according to the manufacturer's instructions. The product was called the GGA1 plasmid library.

The second GGA was performed under the same condition except for another restriction enzyme AarI and Esp3I were used for H-mLibA and B, respectively. The molar ratio of the GGA1 library and the ‘human Gln-tRNA vector’ (The vector and map will be available in addgene) is 1:3, and 50 fmol of the GGA1 library was used. For each library, three 50 ul reactions and one negative control reaction were performed. The transformation, propagation, and plasmid library extraction were performed the same way as the preparation for the GGA1 library. After propagation, colonies were scraped from the plates. Plasmids were extracted using QIAGEN Plasmid Plus Midi Kit (QIAGEN, 12945) according to the manufacturer's instructions.

### CRISPR-Cas9 K562/Jurkat screens

K562 and Jurkat cell lines expressing Cas9 stably and lentivirus pools carrying sgRNA libraries were produced as previously described^14^. K562-Cas9 cells were cultured in 1640 medium with 10% FBS and 1 μg/ml blasticidin on confluency of 0.5 million/ml in shaking incubators at 120 rpm, 37 °C, and with 5% CO2. For sgRNA screening, cells were transduced by lentivirus pools in two biological replicates at a low MOI (~ 0.3). Transduction was performed with enough cells to achieve a representation of at least 500 cells per sgRNA per replicate. After 2 days of culture, transduction efficiency was detected by makte2 fluorescent proteins through flow cytometer. Makte2 positive cells representing × 500 coverage of each sgRNA library were centrifuged and stored at − 80 °C, which were used as starting reference cells (day 0). The other cells were still cultured and selected by puromycin at 2 μg/ml puromycin for the first 4 days and then at 1 μg/ml puromycin for the next 20 days. The same number of sgRNA-expressing cells as day 0 were collected on days 4, 10, 18, and 24, and these time points were marked as day 4, day 10, day 18, and day 24. Jurkat-Cas9 cells were cultured and screened in the same manner as K562, while the cells were collected on days 0, 12, 18, and 24.

### NGS library preparation

Genome DNA was extracted by DNA isolation kit (TIANamp Genomic DNA Kit, Cat. no 4992254) based on the protocols provided by the manufacturer. All genome DNA was used for NGS library construction in the split 50ul PCR reaction as follows: 2 μg DNA template, 0.25 μM forward primer, 0.25 μM reverse primer, 25 μl NEBNext Ultra II Q5 Master Mix (NEB, M0544S) and nuclease-free water up to 50 μl. Before PCR amplification, 20 ul of reaction was used to perform quantitative PCR (qPCR) to quantify the PCR cycle following the program: (1) 98 °C 60 s; (2) 40 cycles of 98 °C 10 s, 65 °C 20 s, 72 °C 20 s; (3) 72 °C 2 min. The final library PCR procedure was the same as qPCR, while the cycles in step (2) were determined according to the results of qPCR. Finally, the PCR product was purified with AMPure XP beads (Beckman, A63882). All primers used here can be found in Supplementary Table 5 (Supplementary Table 5).

### Data analysis

All libraries were sequenced as 150-bp paired-end. The sequencing reads were first undergone adapter removal by ‘cutadapt’. The parameters were set as ‘-n 1 -e 0.1 -O 2 -m 16’^56^. Alignment was conducted using bowtie2 with ‘-np 0 -n-ceil L,0,0.2 –very-sensitive’^57^. Customized references were used according to the sources of the NGS libraries. The successfully aligned reads were assigned to the designed sgRNA-pairs and corresponding genes by a custom Perl script. The read counts were calculated and used for downstream analysis.

To get a reliable human essential gene list, we collected three published essential gene lists^3,12,45^ and three essential gene lists from the DepMap Public 22Q1 dataset including ‘Achilles_common_essentials’, ‘CRISPR_common_essentials’ and ‘common_essentials’. Genes within three of these six lists were summarized as essential genes (EG_1) and were used for downstream analysis. The used non-essential genes were the gold-standard nonessential genes^39^ (NG_1) which were also defined as ‘nonessentials’ in the DepMap Public 22Q1 dataset.

Then, the raw read counts for each library were processed with the BAGEL2^40^, MAGeCk^42^, ScreenBEAM^43^, and PBNA^44^, pipeline. To evaluate the performance of each library, we ran BAGEL2 with default parameters on all replicates, and the essential and nonessential training sets defined above. The parameters for MAGeCk were set as ‘–control-sgrna Negative_control –norm-method median –paired –normcounts-to-file –remove-zero both –sort-criteria pos’. The parameters for ScreenBEAM were set as ‘data.type = 'NGS', do.normalization = TRUE, filterLowCount = TRUE, filterBy = 'control', count.cutoff = 4, nitt = 15,000, burnin = 5000’. We ran PBNA with default parameters on each replicate independently and on all replicates. The results of MAGeCk, ScreenBEAM, and PBNA were used to calculate the AUC value for each sample by pROC^58^ R package.

To identify core essential and cell-type specific essential genes, we ran BAGEL2 with default parameters on day 10, 18, and 24 of K562 samples and day 12, 18, and 24 of Jurkat samples, as these three time points validated the ability to classify essential genes. The algorithm uses certain essential and nonessential gene training sets, and we note that different training sets would get different BF results. So, besides the EG_1 and NG_1 gene lists, we further employed the gold-standard essential genes^39^ (EG_2), the expanded essential genes^45^ (EG_3) by the same group, and another defined non-essential gene list^3^ (NG_2) and paired into four training set combinations: ‘EG_1-NG_1’, ‘EG_1-NG_2’, ‘EG_2-NG_2’ and ‘EG_3-NG_2’. For each cell line, we got 36 gene-level BF results across three libraries (3 time points × 3 libraries × 4 training sets). Based on the Cancer Cell Line Encylcopedia^59^ (CCLE) gene expression data, genes with log TPM < 1 were excluded. Next, to get reliable results, we set five different cutoffs: (1) Genes presented in 24 results with BF ≥ 6 or any results with BF ≥ 20; (2) Genes presented in 24 results with BF ≥ 6 or any results with BF higher than three-sigma above the mean; (3) Genes presented in 18 results with BF ≥ 6 or any results with BF higher than three-sigma above the mean; (4) Genes presented in 24 results of K562 and presented in 16 results of Jurkat with BF ≥ 6 or any results with BF higher than three-sigma above the mean; (5) Genes presented in 24 results of K562 and presented in 18 results of Jurkat with BF ≥ 6 or any results with BF higher than three-sigma above the mean. A gene with BF above the threshold that hits three cutoffs was defined as effectively essential.

### Supplementary Information

Supplementary Information 1.

Supplementary Information 2.

Supplementary Information 3.

Supplementary Information 4.

Supplementary Information 5.

Supplementary Information 6.

Supplementary Information 7.

## Data Availability

All raw and processed sequencing data generated in this study have been submitted to the NCBI Gene Expression Omnibus (GEO; https://www.ncbi.nlm.nih.gov/geo/) under accession number GSE223086.
