# Author Correction: Optimized minimal genome-wide human sgRNA library

**DOI:** 10.1038/s41598-023-40304-4

**Published:** 2023-08-16

**Authors:** Yangfan Zhou, Lixia Wang, Zhike Lu, Zhenxing Yu, Lijia Ma

**Affiliations:** 1 College of Life Sciences, Zhejiang University, Hangzhou, 310058 China; 2 School of Life Sciences, Westlake University, 600 Dunyu Road, Hangzhou, 310030 Zhejiang China; 3 Institute of Biology, Westlake Institute for Advanced Study, Hangzhou, 310024 Zhejiang China; 4 Westlake Laboratory of Life Sciences and Biomedicine, Hangzhou, 310024 Zhejiang China; 5 School of Life Sciences, Fudan University, Shanghai, 200433 China

Correction to: *Scientific Reports* 10.1038/s41598-023-38810-6, published online 18 July 2023

The original version of this Article contained an error in the Data Availability section,

“All raw and processed sequencing data generated in this study have been submitted to the NCBI Gene Expression Omnibus (GEO; https://www.ncbi.nlm.nih.gov/geo/) under accession number GSE223086. To review GEO accession GSE223086: Go to https://apc01.safelinks.protection.outlook.com/?url=https%3A%2F%2Fwww.ncbi.nlm.nih.gov%2Fgeo%2Fquery%2Facc.cgi%3Facc%3DGSE223086&data=05%7C01%7Cmalabdata%40westlake.edu.cn%7C161fec6e4bfb4b17f8d108db0e6cf43a%7C7e82de2f7ef644169b9644c1457be81b%7C1%7C0%7C638119633354408598%7CUnknown%7CTWFpbGZsb3d8eyJWIjoiMC4wLjAwMDAiLCJQIjoiV2luMzIiLCJBTiI6Ik1haWwiLCJXVCI6Mn0%3D%7C3000%7C%7C%7C&sdata=WxsnnR%2BLiJgAQ5Cxqfd7jkgL8LJ%2FkqDfcUM42vNoYx4%3D&reserved=0. Enter token of mnymuqxhwhbmv into the box.”

now reads:

“All raw and processed sequencing data generated in this study have been submitted to the NCBI Gene Expression Omnibus (GEO; https://www.ncbi.nlm.nih.gov/geo/) under accession number GSE223086.”

The original Article has been corrected.

