# Supplementary material for: Optimized minimal genome-wide human sgRNA library
Source: Sci Rep. 2023 Jul 18;13:11569. doi: 10.1038/s41598-023-38810-6 (PMC10354020; doi:10.1038/s41598-023-38810-6)
Supplement: Supplementary file 1 — Supplementary Information 1. [file 41598_2023_38810_MOESM1_ESM.pdf]

Supplementary Fig. 1

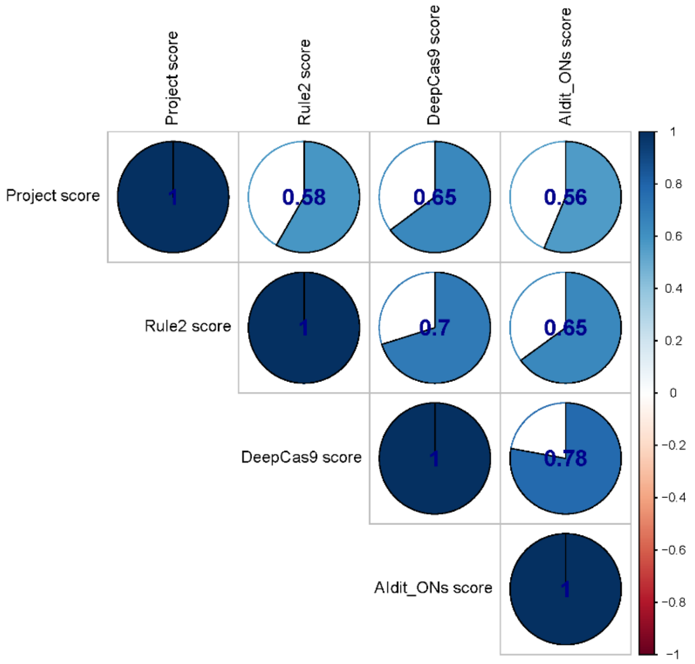

The Pearson correlations between Project scores, Rule2 scores, DeepCas9 scores, and AIdit_ONs scores. These scores were calculated based on the sgRNA sequence in the primary pool.

Supplementary Fig. 2

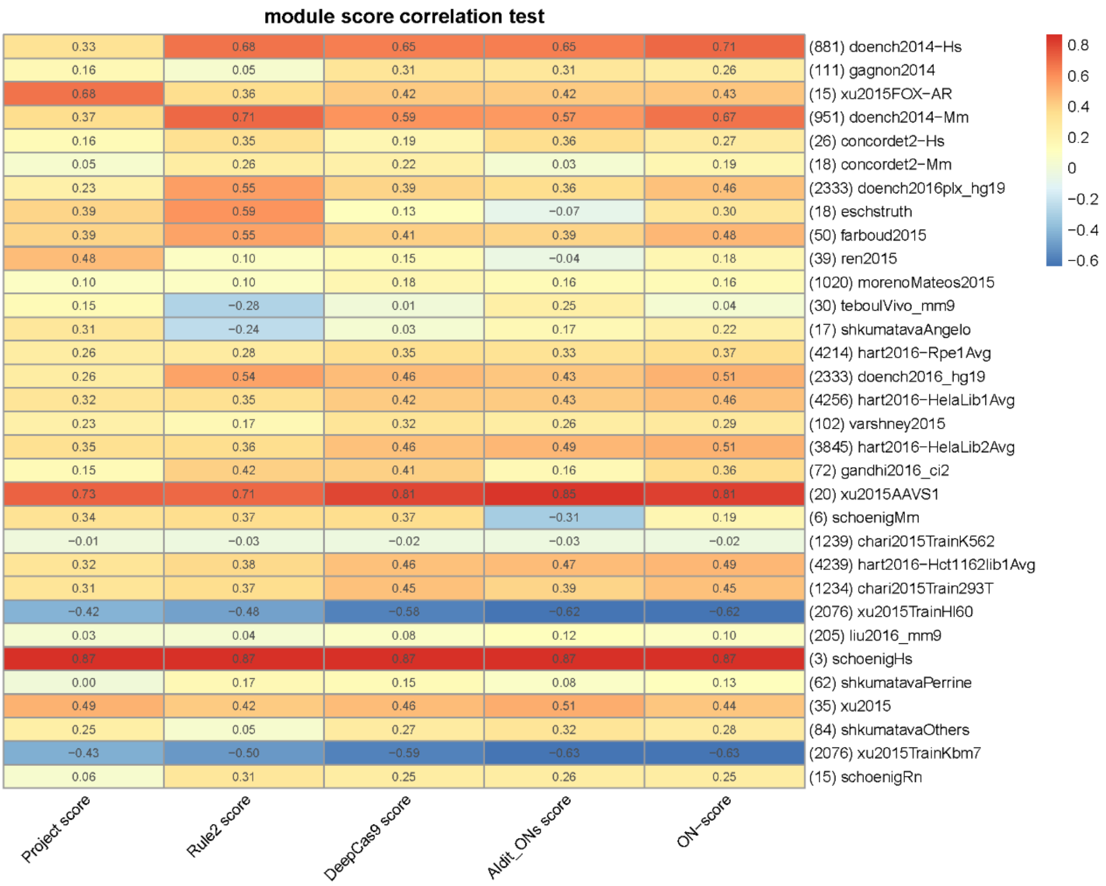

Heatmap of Spearman rank correlation coefficients between prediction scores and published datasets. The name of prediction scores is shown along the horizontal axis, the sgRNA number, and the name of each dataset on the vertical. The higher this value is, the better the prediction.

Supplementary Fig. 3

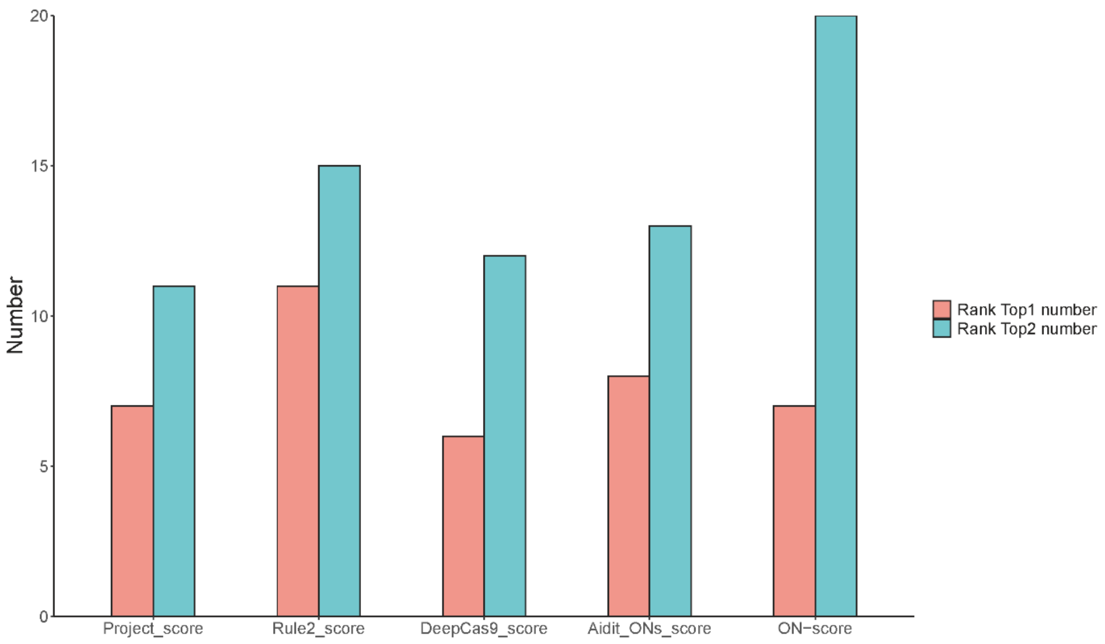

Summary of the performance of different prediction scores across 32 published datasets. For each dataset, prediction scores were ranked according to the corresponding correlation coefficient between predicted efficiency and obtained cleavage efficiency. Rank Top1 represents the prediction score that has the best performance, rank Top2 represents the prediction score that has the best or second-best performance.

Supplementary Fig. 4

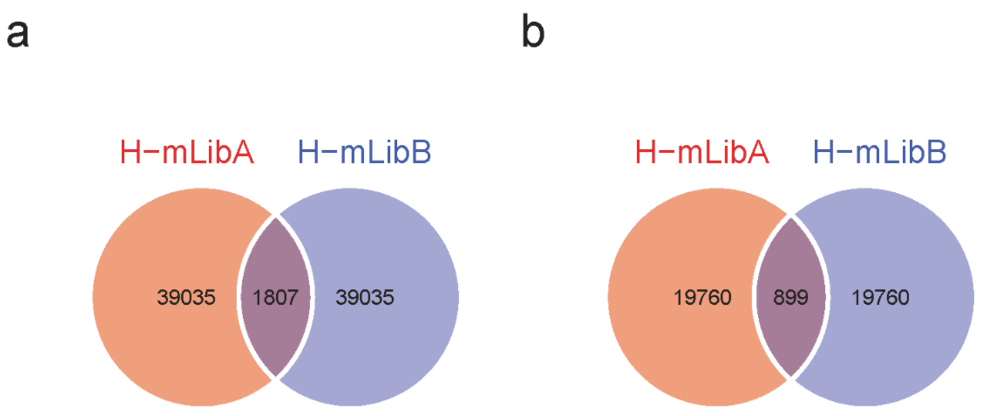

Shared sgRNAs (**a**) and sgRNA pairs (**b**) of H-mLibA and H-mLibB. Both H-mLibA and H-mLibB contain 20,659 gene-targeted sgRNA pairs, and each contains 40,842 unique sgRNAs.

Supplementary Fig. 5

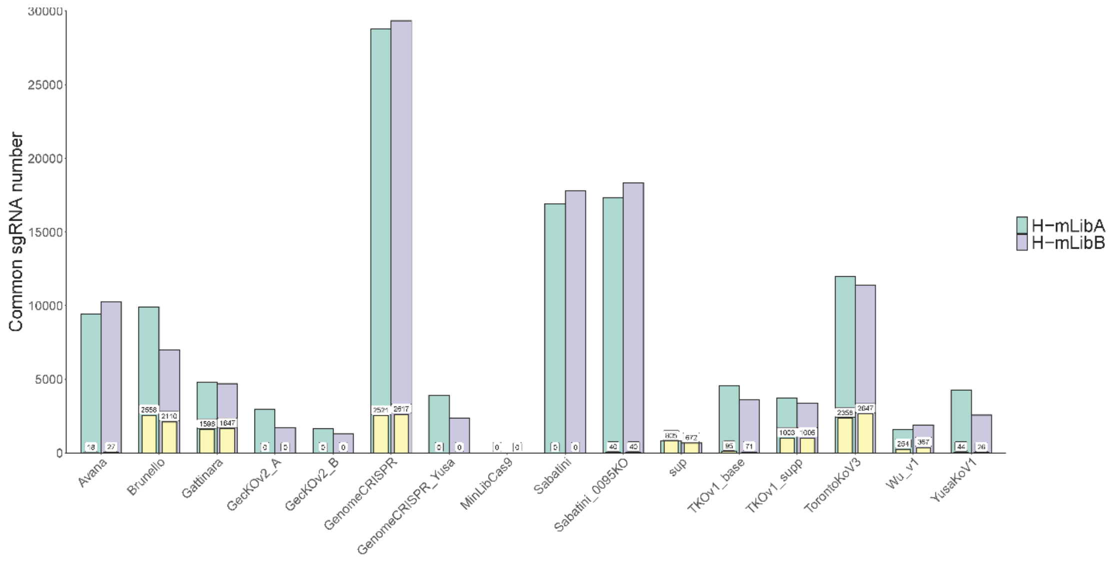

sgRNAs of H-mLibA or H-mLibB shared with other reported sgRNA libraries. The colored yellow bars represent the unique consensus sgRNAs in each library and the exact numbers are

shown in the text box. H-mLibA and H-mLibB contain sgRNAs that were consistent with other libraries, due to the different sgRNA selection strategies, the amount of the same sgRNA is different.

Supplementary Fig. 6

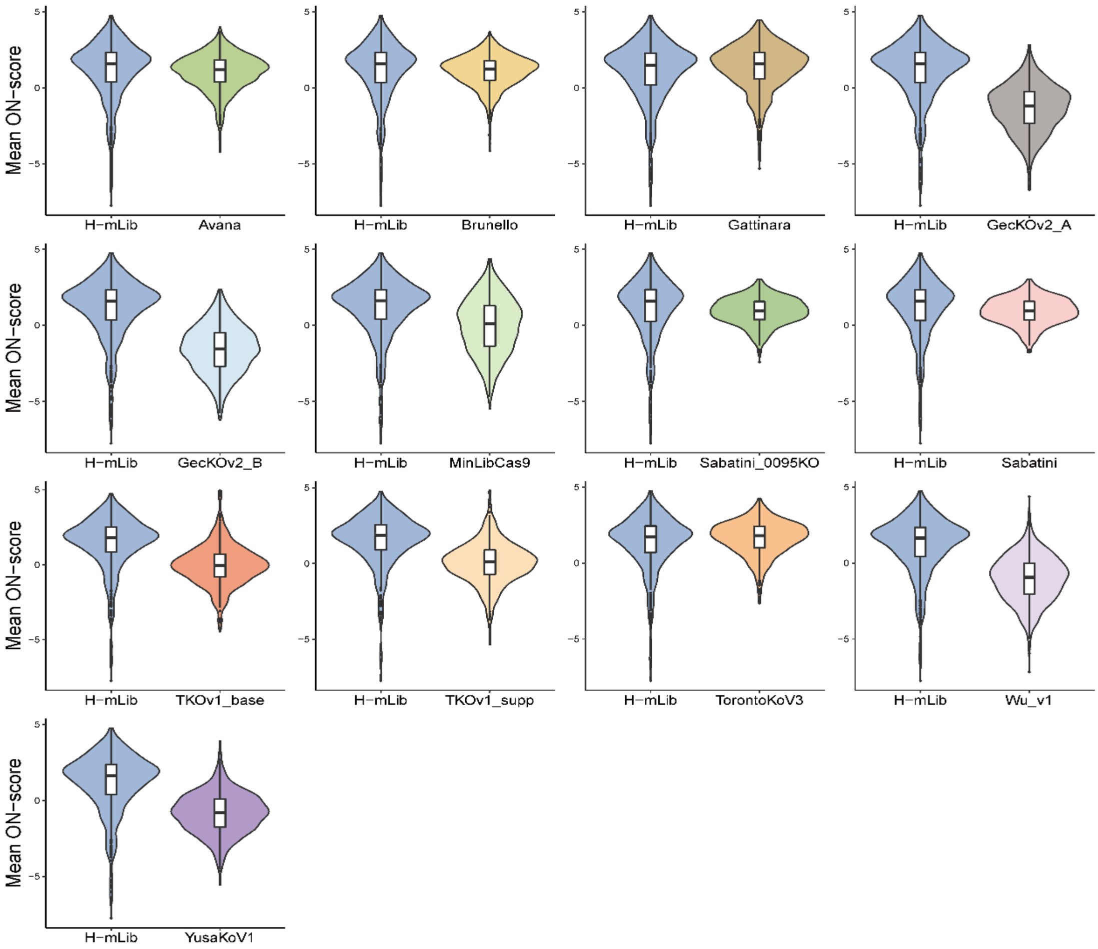

The ON-score distributions of "sup" sgRNAs in H-mLib compared with the same gene target sgRNAs in other libraries. The median value of each compare group: Avana 1.21 and H-mLib 1.60, Brunello 1.23 and H-mLib 1.59, Gattinara 1.58 and H-mLib 1.48, GecKOv2_A -1.20 and H-mLib 1.59, GecKOv2_B -1.53 and H-mLib 1.59, MinLibCas9 0.13 and H-mLib 1.62,

Sabatini_0095KO 0.97 and H-mLib 1.59, Sabatini 0.96 and H-mLib 1.59, TKOv1_base -0.04 and H-mLib 1.81, TKOv1_supp 0.09 and H-mLib 1.84, TorontoKoV3 1.79 and H-mLib 1.74, Wu_v1 -0.94 and H-mLib 1.64, YusaKoV1 -0.78 and H-mLib 1.62.

Supplementary Fig. 7

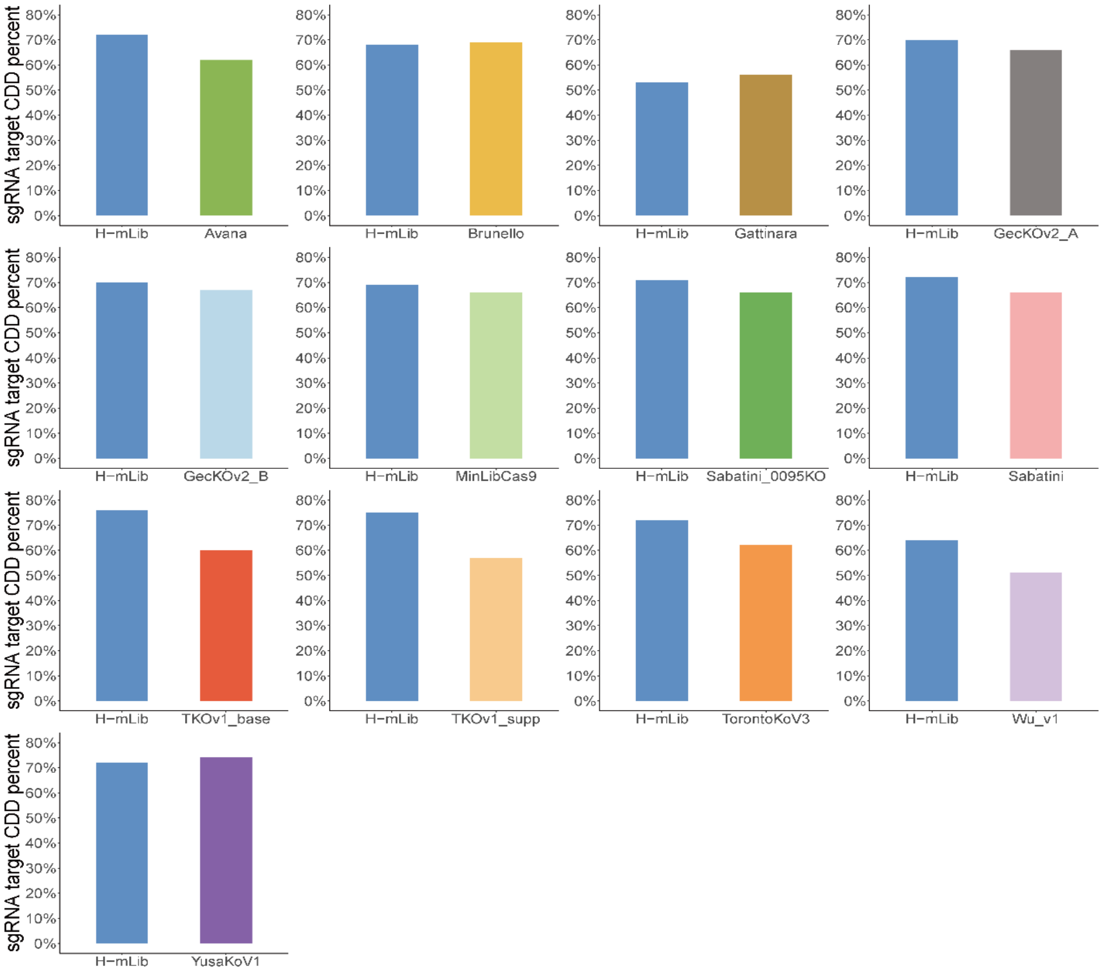

The percent of sgRNA target CDD of “sup” sgRNAs in H-mLib compared with the same gene target sgRNAs in other libraries. H-mLib shows higher CDD target percent compared with most libraries (n=10), only Brunello (69% to 68%), Gattinara (56% to 53%), and YusaKoV1 (74%

to 72%) were slightly higher.

Supplementary Fig. 8

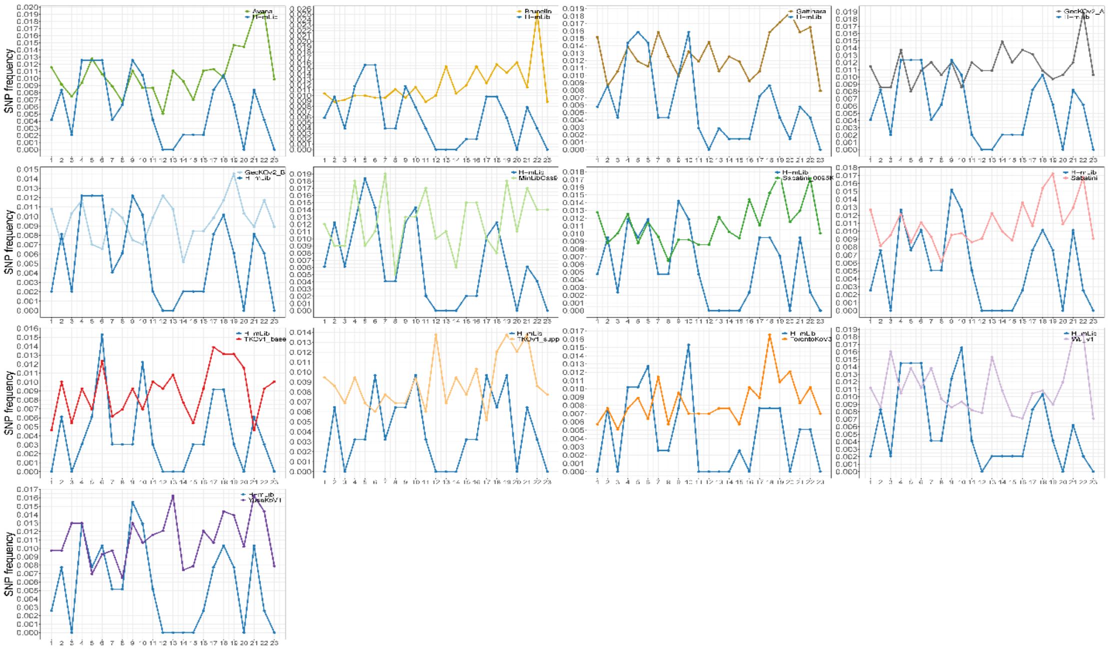

SNP frequency at each site of sgRNA (1-20) and PAM (21-23) sequence of “sup” sgRNAs in H-mLib compared with the same gene target sgRNAs in other libraries. Frequency equal to 0 means there is no SNP in the sgRNA set.

Supplementary Fig. 9

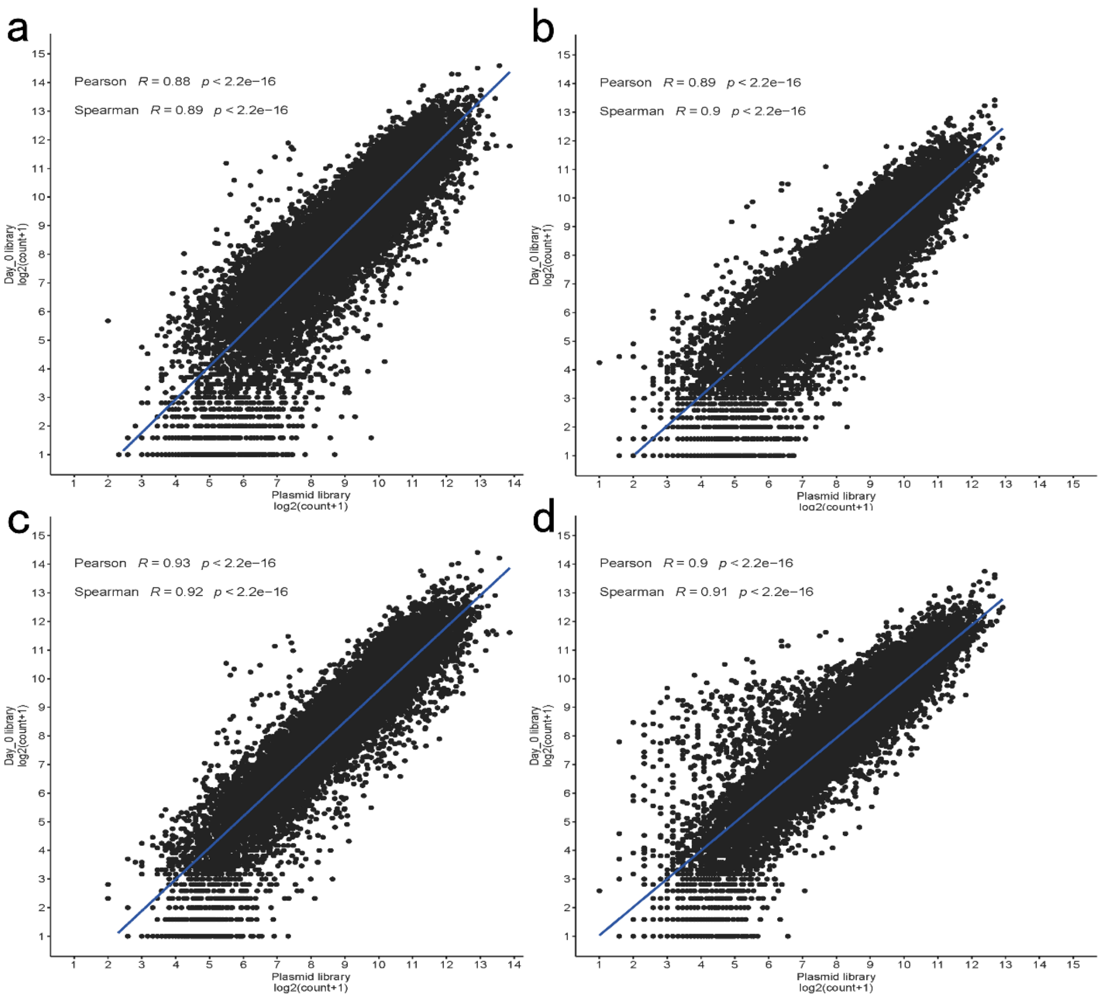

Library quality control before and after the lentivirus vector packaging. The scatterplots in panels (a) and (b) present the correlation of sgRNA counts between the plasmid library and the corresponding "0 day" library of H-mLibA and H-mLibB in K562 cells. The scatterplots in panels (c) and (d) present the correlation of sgRNA counts between the plasmid library and the corresponding day 0 library of H-mLibA and H-mLibB in Jurkat cells. The correlation coefficients between 0.88 and 0.93 indicate a positive correlation.

Supplementary Fig. 10

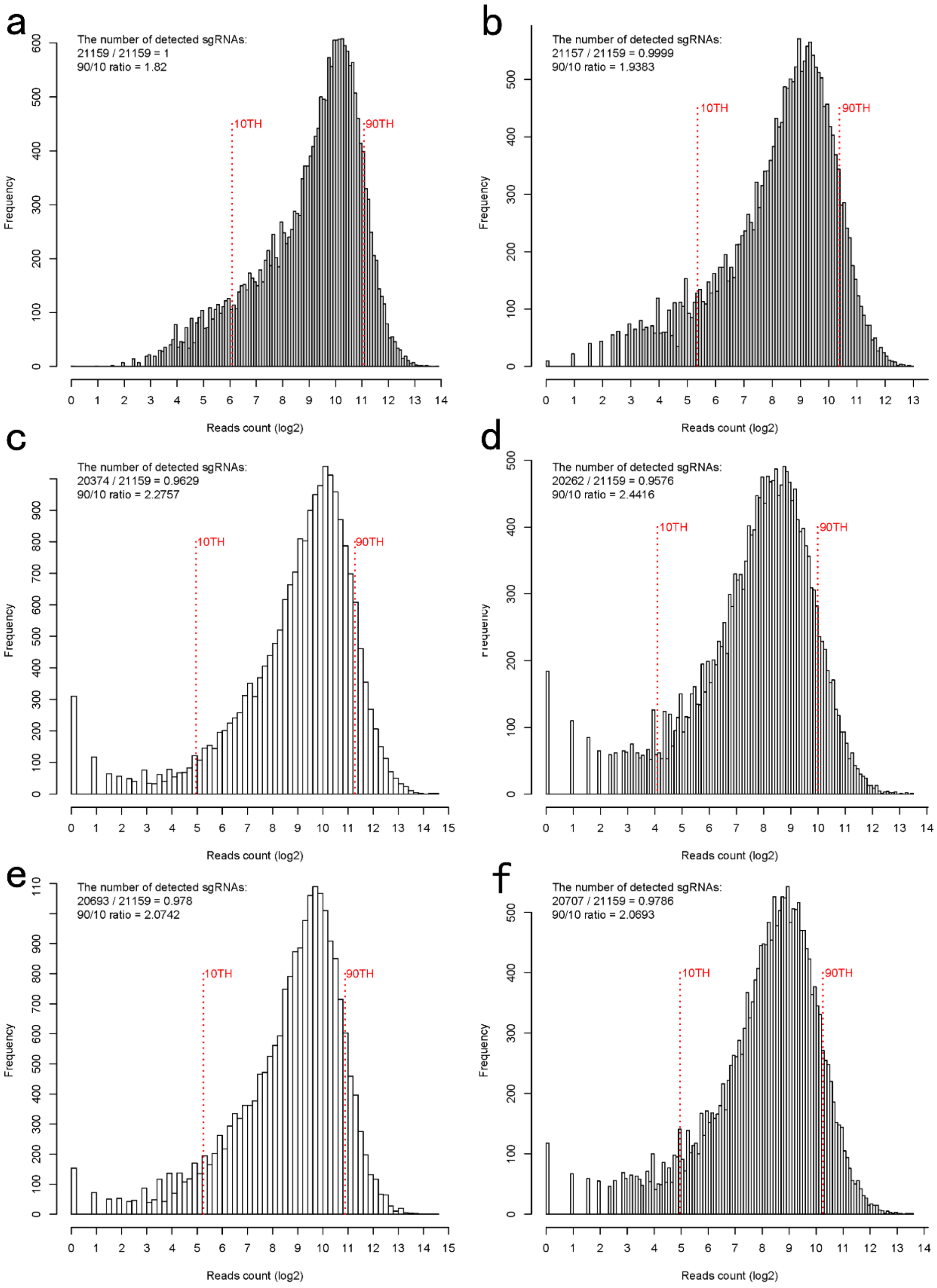

sgRNA Count frequency distribution and coverage. The histograms in panels (a) and (b) present the frequency distribution of sgRNA counts in the H-mLibA and H-mLibB plasmid libraries,

respectively. The plasmid libraries covered 100% and 99.99% of sgRNAs of H-mLibA and H-mLibB, respectively. In K562 cells, the coverage of sgRNA in the H-mLibA (c) and H-mLibB (d) “0 day” library was 96.29% and 95.76%, respectively. In Jurkat cells, the coverage of sgRNA in the H-mLibA (e) and H-mLibB (f) “0 day” library was 97.80% and 97.86%, respectively. The ratio of sgRNA counts at the 90th percentile and the 10th percentile for each of the plasmid libraries and the “0 day” libraries, and the ratios are 1.82-2.44, indicating good uniformness of sgRNAs.

Supplementary Fig. 11

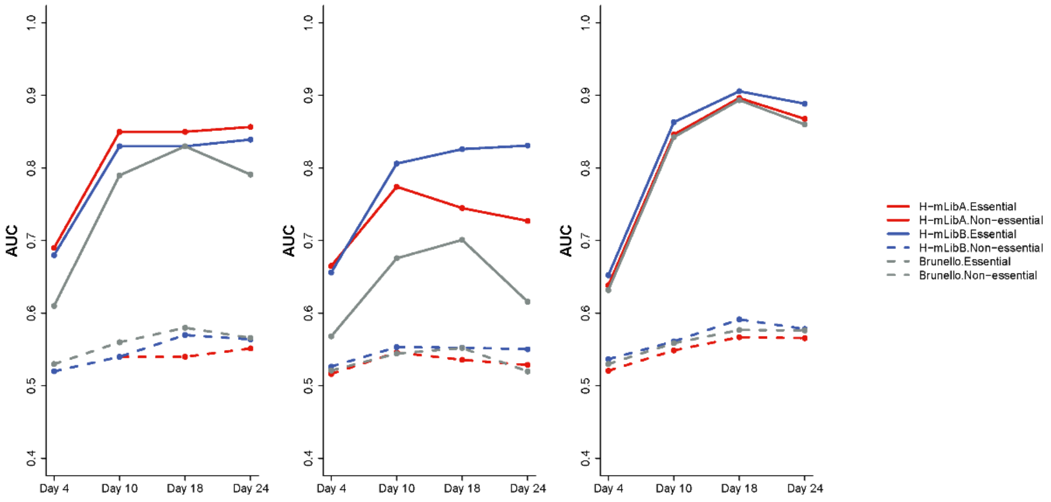

Area under the curve (AUC) analysis of individual sgRNAs targeting essential (dotted solid line) and non-essential (dotted dash line) gene sets in the H-mLibA/B and Brunello library screening data at different time points. The results were calculated by MAGeCK (left), ScreenBEAM (middle), and PBNA (right).

Supplementary Fig. 12

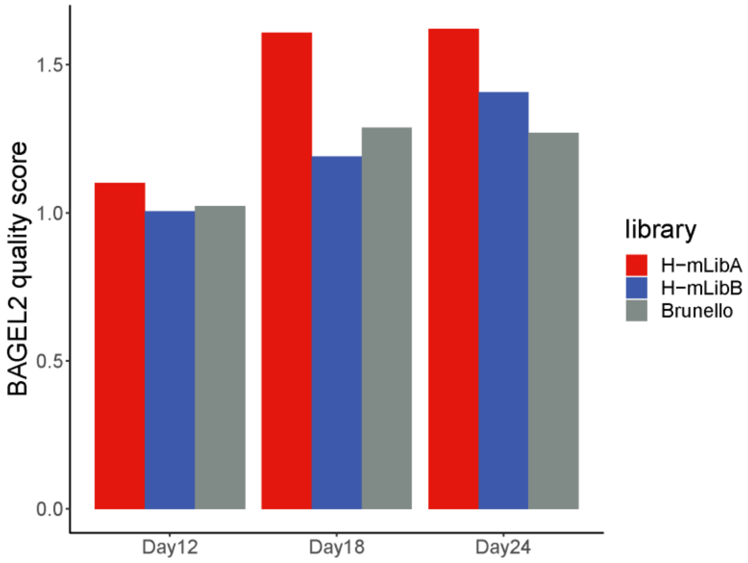

The BAGEL2 quality scores were calculated at Day12, Day18, and Day24 of the Jurkat screening by H-mLibA, H-mLibB, and Brunello CRISPR libraries.

Supplementary Fig. 13

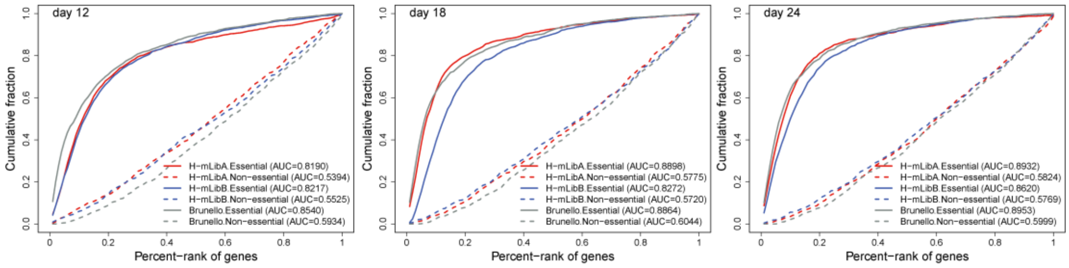

ROC-AUC analysis of each library at different time points in the Jurkat cell line. The ROC-AUC curves show the MAGeCK results for individual sgRNAs targeting essential (solid line) and non-essential (dashed line) gene sets in the H-mLibA, H-mLibB, and Brunello libraries screened in Jurkat cells at time points day 12, day 18, and day 24. On day 12, all three libraries

demonstrate similar performance. On day 18 and day 24, H-mLibA performs similarly to the Brunello library while exhibiting the highest AUC score.

Supplementary Fig. 14

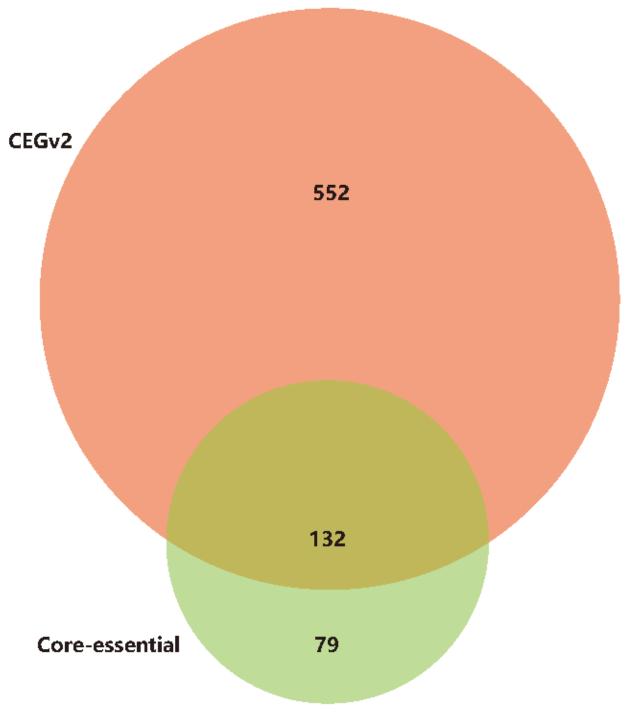

Expanded human core-essential gene number. CEGv2 (n=684) is the gold-standard core-essential gene list [46]. Core-essential (n=211) is identified by H-mLib and Brunello libraries.

Supplementary Fig. 15

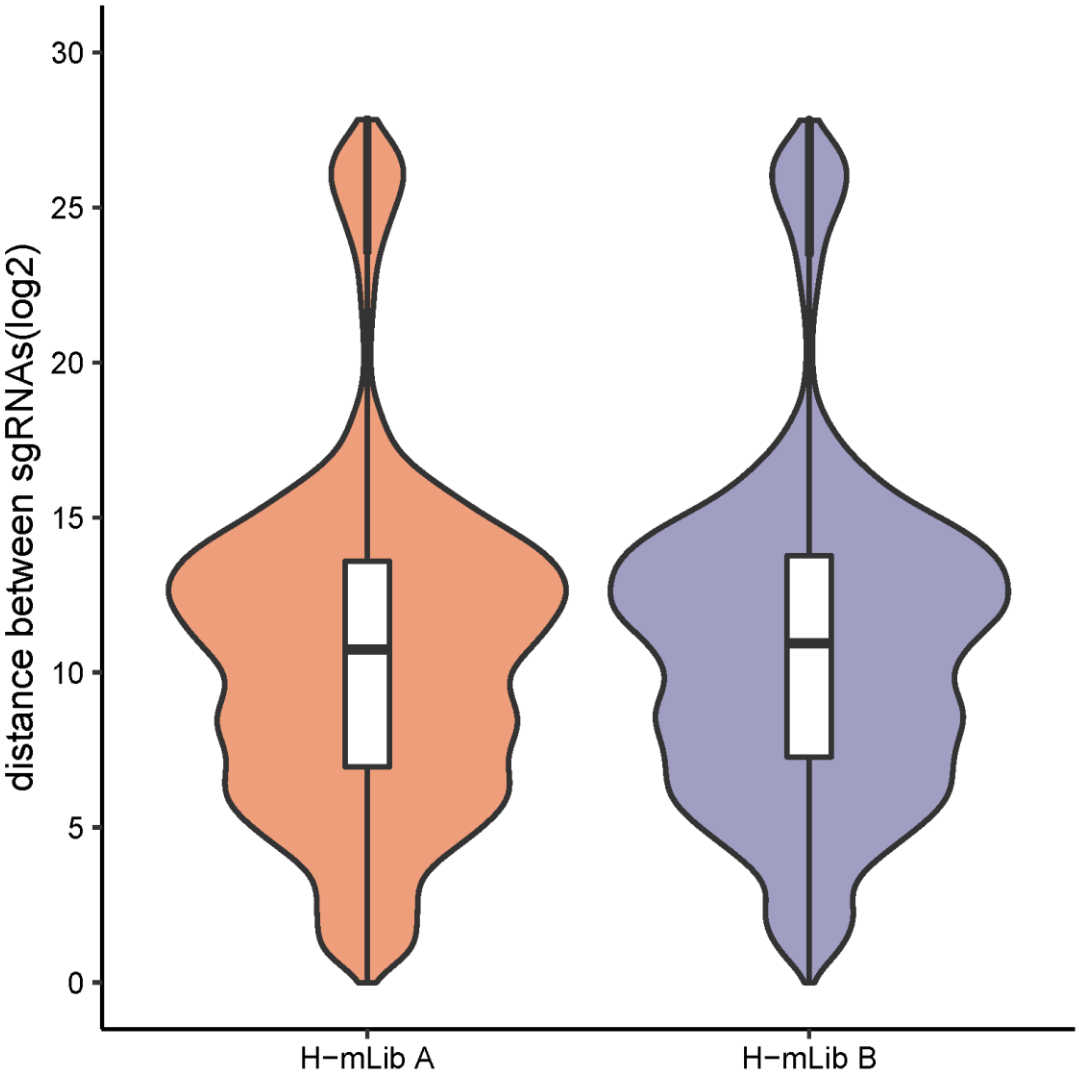

The distance between two sgRNA in the H-mLib library. The median distance of H-mLib A and H-mLib B are 1,701 and 1,967, respectively.

Supplementary Fig. 16

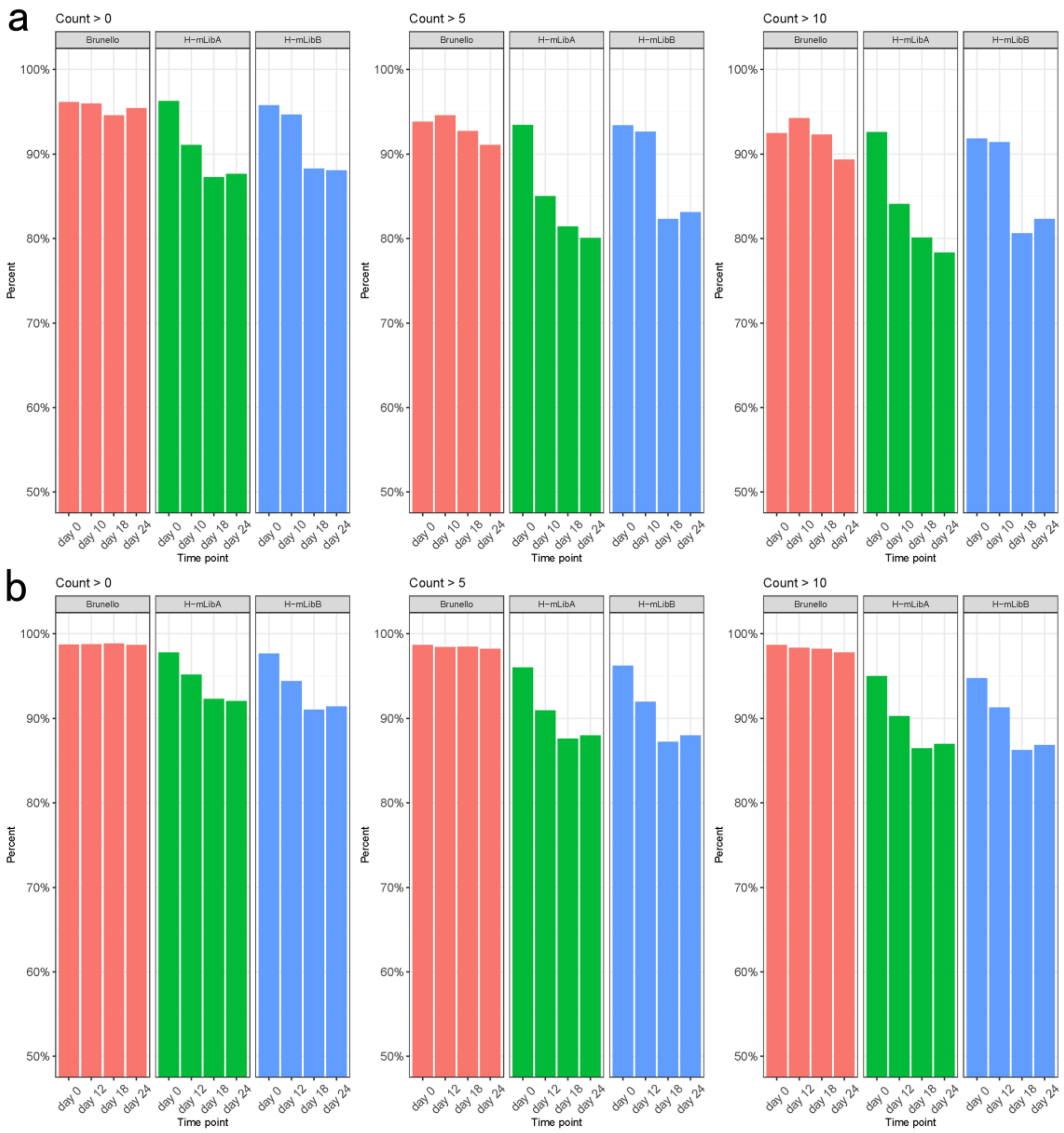

The gene detection percentages of each library at different time points in the K562 and Jurkat cell lines. The histograms present the gene detection percentages of three levels (count > 0, count > 5, and count >10) in K562 (a) and Jurkat (b) screens at different time points. In K562 cells, at the count > 0 levels, over 95% of genes were detected at any time point in Brunello, while in both H-mLibA and H-mLibB, the percentages ranged from 96% to 88%; At the count >

5 levels, in Brunello, the percentages ranged from 94% to 91%, while in H-mLibA and H-mLibB the percentages ranged from 93% to 80% and 93% to 83%, respectively; At the count > 10 levels, in Brunello, the percentages ranged from 94% to 89%, while in H-mLibA and H-mLibB, the percentages ranged from 93% to 78% and 92% to 80%, respectively. In Jurkat cells, at any level, over 98% of genes were detected at any time point in Brunello. At the count > 0 levels, in H-mLibA and H-mLibB, the percentages ranged from 98% to 92% and 98% to 91%, respectively; At the count > 5 levels, in H-mLibA and H-mLibB, the percentages ranged from 96% to 88% and 96% to 87%, respectively; At the count > 10 levels, in both H-mLibA and H-mLibB, the percentages ranged from 95% to 86%.
